# Quality Improvement Initiative Enhances Outpatient Pediatric Pulmonology Follow-up for Premature Infants with Bronchopulmonary Dysplasia

**DOI:** 10.1097/pq9.0000000000000736

**Published:** 2024-06-07

**Authors:** Eliaz Brumer, Sanjiv Godse, Leela Chandrasekar, Tuba Kockar Kizilirmak, Eleanor Blythe, Yeisid Gozzo, Steven Peterec, Sarah Kandil, Matthew Grossman, Laura Chen, Pnina Weiss, Beverley Sheares

**Affiliations:** * From the Department of Pediatrics, Section of Respiratory, Allergy-Immunology, and Sleep Medicine, Yale University, New Haven, Conn.; † Department of Pediatrics, Section of Neonatal-Perinatal Medicine, Yale University, New Haven, Conn.; ‡ Department of Pediatrics, Section of Critical Care Medicine, Yale University, New Haven, Conn.; § Department of Pediatrics, Section of General Pediatrics, Yale University, New Haven, Conn.

## Abstract

**Introduction:**

Bronchopulmonary dysplasia (BPD) is a chronic lung disorder affecting many premature infants. Infants with BPD have higher hospital readmission rates due to respiratory-related morbidity. We aimed to increase the rates of outpatient pulmonary follow-up and attendance of premature babies with moderate and severe BPD to above 85% within 6 months.

**Methods:**

We conducted a quality improvement project at Yale New Haven Children’s Hospital. Key interventions included developing a BPD clinical pathway integrated into the electronic medical record to assist providers in correctly classifying BPD severity, assigning the appropriate International Classification of Diseases, 10th Revision code (P27.1), and providing standardized treatment options. The outcome measures included correct diagnosis and classification of BPD, the percentage of patients with BPD scheduled for pediatric pulmonology appointments within 45 days, and the percentage attending those appointments.

**Results:**

There were 226 patients in our study, including 85 in the baseline period. Correct diagnosis of BPD increased from 49% to 95%, the percentage of scheduled appointments increased from 71.9% to 100%, and the percentage of appointments attended increased from 55.6% to 87.1%.

**Conclusions:**

Our quality improvement initiative improved the accuracy of diagnosis, severity classification, and outpatient pulmonary follow-up of children with moderate and severe BPD.

## INTRODUCTION

**Fig. 1.**
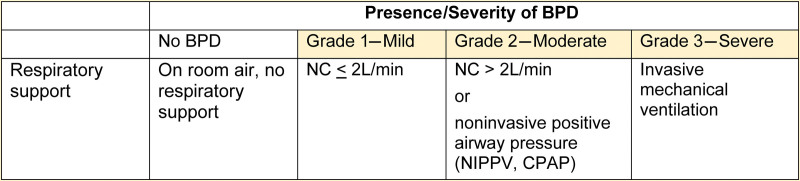
BPD definition and severity as recommended by Jensen et al.^5^ CPAP, continuous positive airway pressure; NIPPV, nasal intermittent positive pressure ventilation.

Bronchopulmonary dysplasia (BPD) is the most common form of chronic lung disease, primarily affecting premature infants. It is thought to be the result of injury to the developing lung.^1–3^ Defining BPD continues to be challenging, and different criteria have been used in the literature. Shennan et al^4^ suggested a definition for BPD, which correlated supplemental oxygen use at 36 weeks of corrected gestational age (CGA) with respiratory comorbidity at 2 years of age. Since then, less invasive ventilatory strategies, such as early continuous positive airway pressure and high-flow nasal cannula, have been incorporated into the standard treatment of premature infants. Together with less invasive methods of surfactant administration, improved nutritional support, and strategies aimed to minimize ongoing lung injury, these approaches have improved the clinical course and outcomes of the postnatal lung development of premature newborns.^1,2^ Jensen et al.^5^ recommended new criteria for defining BPD. Grades 1, 2, and 3 correspond to mild, moderate, and severe BPD (Fig. 1), based on respiratory support at 36 weeks of CGA. They demonstrated that this definition better predicts adverse neurodevelopmental and pulmonary outcomes.^1–3^

Outpatient management of infants with BPD often varies, and infants with BPD require frequent readmission to the hospital in the first 2 years after birth for respiratory-related problems.^1,2,6,7^ BPD is associated with long-term respiratory morbidity and increased risk of developing chronic respiratory conditions, such as asthma-like symptoms, exercise intolerance, and pulmonary hypertension during childhood or adulthood.^1,7–9^ Despite these associations, there is a lack of consistency in diagnostic, therapeutic, and care practices among various healthcare providers.^8,10,11^ Inaccurate diagnosis of BPD may result in inadequate monitoring and management of respiratory symptoms, possibly leading to worsening lung disease, respiratory distress, or other complications, which can impact the infant’s overall health outcomes and increase mortality risk.^1,8,9,11,12^

The management of infants with BPD can be a challenging task. Within our institution, there was variation in management among neonatal intensive care (NICU) and pulmonary providers during the hospital stay and long-term care. Despite efforts to ensure adequate follow-up care, it was unclear how many infants with BPD were lost to follow-up after discharge. Given the growing body of literature supporting the association of BPD with long-term comorbidities, there is a pressing need to improve follow-up care for these infants. To this end, we aimed to increase the rate of scheduled pulmonology outpatient appointments for infants with moderate or severe BPD within 45 days of initial hospital discharge. Additionally, we aimed to achieve an attendance rate of more than 85% for pulmonology outpatient appointments within 6 months for all infants diagnosed with moderate or severe BPD.

## METHODS

### Context

We conducted a quality improvement project from January 2021 to September 2023 at Yale New Haven Children’s Hospital, an academic medical center with approximately 4,500 births per year that provides care for more than 1,000 critically ill newborns annually. In our institution, premature infants are placed in two units (acute and chronic), where they receive standardized care, which is provided under guidelines by the NICU team. Before this project, BPD was diagnosed based on the National Institutes of Health 2001 definition^12^; however, the follow-up with pulmonary teams was not standardized and depended on the NICU team’s decision.

**Fig. 2.**
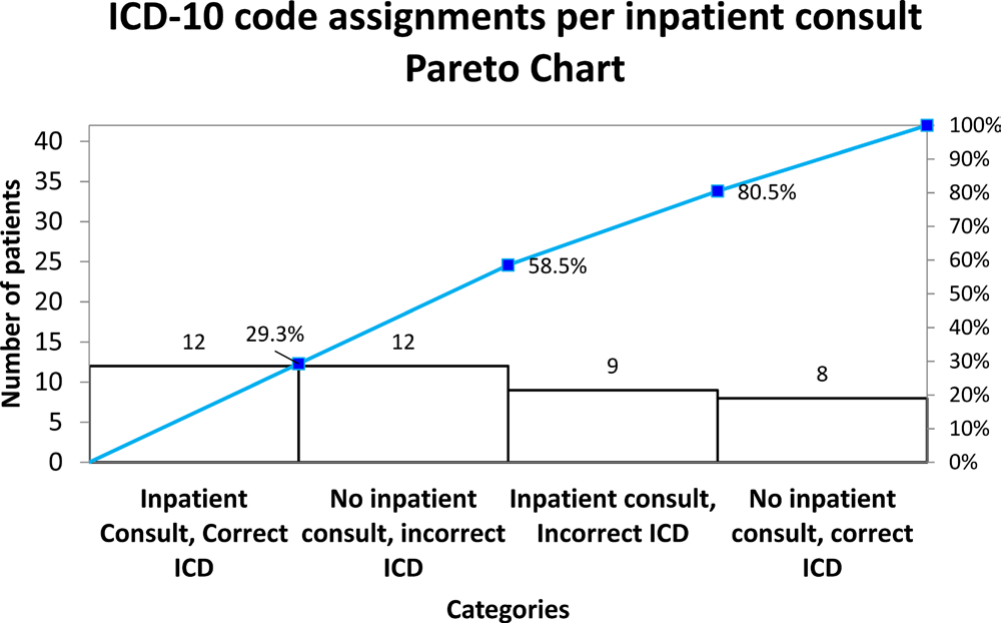
Pareto chart for the 41 BPD patients during the baseline period from January 21 to December 21.

We included all infants discharged from the Yale New Haven Children’s Hospital NICU with a diagnosis of moderate or severe BPD. Criteria for diagnosis were based on the 2019 definition of BPD by Jensen et al^5^ (Fig. 1). We excluded patients who did not meet moderate or severe BPD criteria, expired, or were transferred to another healthcare facility. Because there was no prior standardized approach to identify infants with BPD, during the baseline period (January 2021–December 2021), we compiled a dataset of infants who were thought to have BPD using electronic medical records (EMRs, Epic Systems, Verona, Wis.). This list included infants discharged from the NICU labeled with any International Classification of Diseases, 10th Revision (ICD-10) codes suggestive of respiratory disorders [eg, chronic lung disease of prematurity (P27.9), chronic lung disease due to disorders of surfactant (J84.83), and bronchopulmonary dysplasia (P27.1)] and premature infants who were discharged home on oxygen and/or diuretic treatment regardless of their ICD-10 codes. The list was then manually screened by reviewers (E.B., S.G., and T.K.K., 86 patients, averaging 10 min per patient) to ascertain whether the identified patients met the 2019 definition of BPD by Jensen et al.^5^ and to retrospectively assign an agreed ICD-10 code (P27.1) and a severity classification. We continued monitoring this dataset throughout our entire project. In total, we examined the medical records of 226 patients. Our baseline period data showed that 51.2% of ICD-10 code assignments were incorrect, independent of pediatric pulmonology consultation (Fig. 2).

Additionally, we determined the proportion of patients with moderate and severe BPD (based on the Jensen criteria) who were scheduled for a pulmonary follow-up appointment and completed their appointment within 45 days of home discharge.

### Intervention

**Fig. 3.**
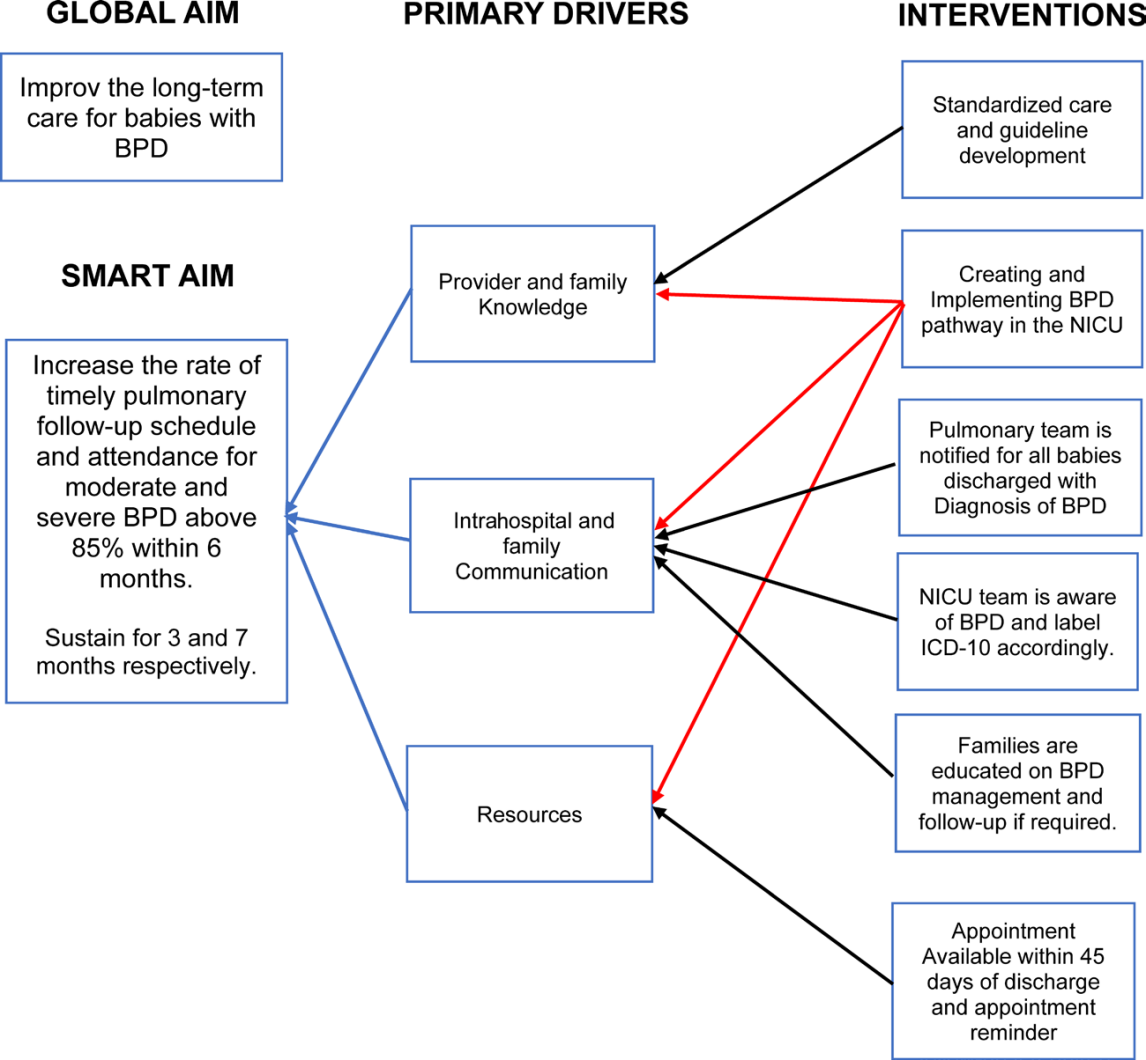
Key driver diagram.

Our intervention period was divided into two distinct phases. During the first phase (January 2022 to September 2022), we formed a multidisciplinary team that included NICU physicians and nurses, hospital pharmacists, and pediatric pulmonologists. We identified three key drivers (Fig. 3): (1) provider and family knowledge; (2) communication between the pediatric pulmonology team, NICU team, and families; and (3) available resources that could impact accurate BPD diagnosis, classification, and timely pulmonary follow-up. During the project, we implemented six interventions (Fig. 3) to increase the rate of scheduled and completed appointments for patients with moderate or severe BPD. During the second phase (September 2022–September 2023), we implemented the BPD pathway in the EMR.

#### Provider and Family Knowledge

##### Standardized Care and Guideline Development.

 During this period, the Department of Pediatric Pulmonology reviewed the literature on managing premature infants diagnosed with BPD. A standardized guideline was then developed and presented to the NICU department for consideration. The guideline underwent several iterations before publication. This guideline was the template for the BPD pathway.

##### Creating and Implementing BPD Pathway in the NICU.

**Fig. 4.**
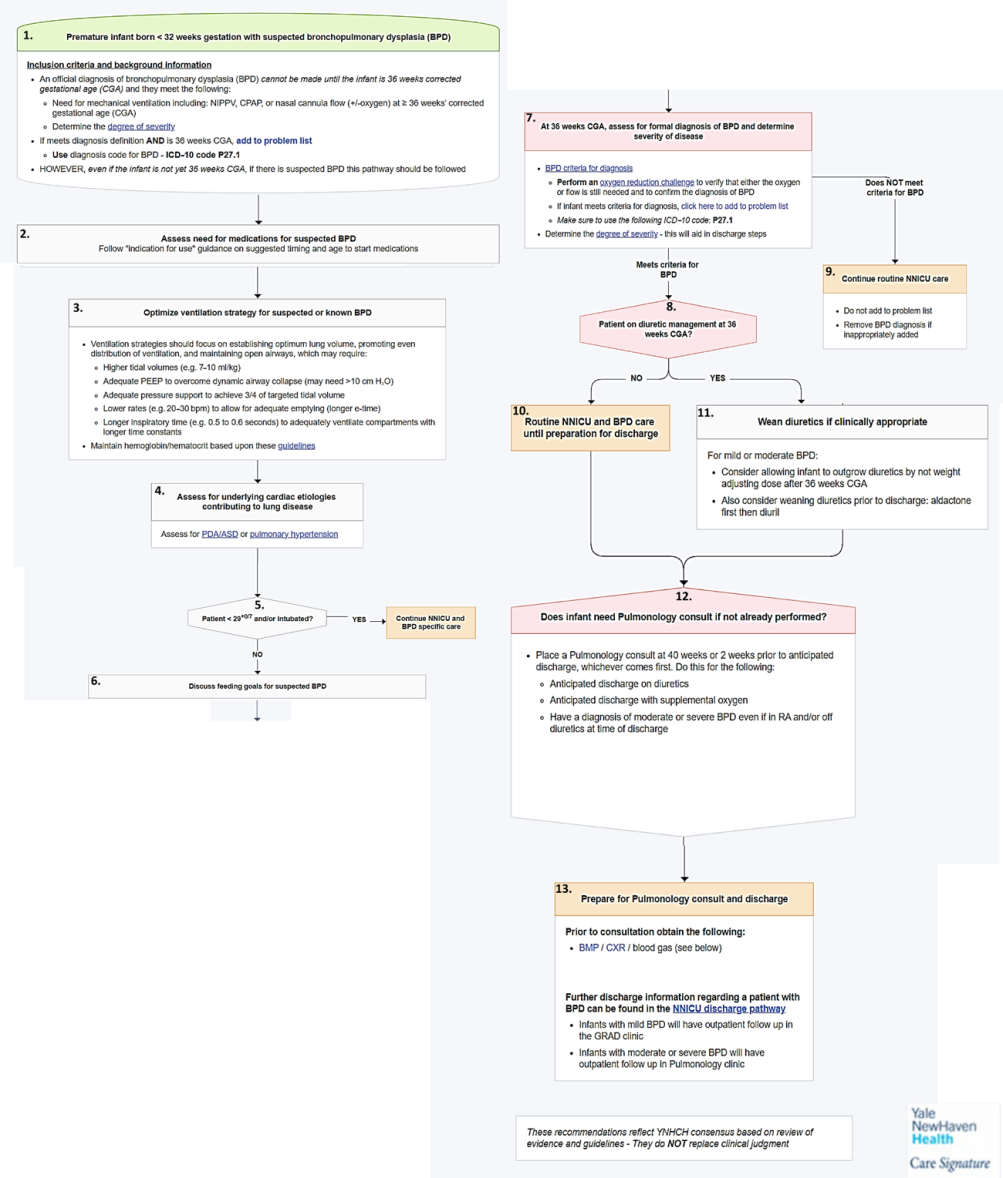
BPD pathway. ASD; BMP; CXR; GRAD; NNICU; PDA; PEEP; RA; YNHCH, Yale New Haven Children’s Hospital. ASD, atrial septal defect; BMP, basic metabolic panel; CXR, chest x-ray; GRAD, graduate; NNICU, neonatal intensive care unit; PDA, patent ductus arteriosus; PEEP, positive end-expiratory pressure; RA, room air.

 As part of a system-wide initiative, the Care Signature program, an electronically integrated clinical pathway was developed (Fig. 4) with a multidisciplinary team comprising neonatologists, nurses, pediatric pulmonologists, and pharmacists. The pathway was designed through a consensus method, ensuring collaborative decision-making, incorporating evidence-based best practices, and standardizing key processes based on the developed standardized guideline. These processes included the appropriate timing of BPD diagnosis, assignment of ICD-10 codes (P27.1), effective medication management and ventilation strategies to establish optimum lung volume and promote even ventilation distribution (Box 3, Fig. 4). Standard orders were integrated into the pathway to facilitate streamlined clinical decision-making, including a recommendation for pediatric pulmonology consultation, laboratory testing and imaging, and a link for BPD discharge information (Boxes 12 and 13, Fig. 4). The utilization of the pathway serves as a process measure monitored throughout the project.

In September 2022, the pathway was linked to suggestion criteria within the Epic electronic health record system (Epic Systems), such that a pathway link would seem in the storyboard for any patient (1) with any prior BPD diagnosis, or (2) born at less than 32 weeks gestation and at least 36 weeks of CGA. The EPIC storyboard configuration is repeatedly triggered for each patient for one week, starting at 36 weeks CGA. Throughout this week, it consistently displays the BPD pathway suggestion, accessible to any healthcare provider who wishes to utilize it.

#### Intrahospital and Family Communication

##### The Pulmonary Team Is Notified of All Babies Discharged with the Diagnosis of BPD.

 The Care Signature BPD pathway included a crucial reminder to officially consult pediatric pulmonology at CGA 40 weeks or 2 weeks before anticipated discharge (Box 12, Fig. 4) and schedule a postdischarge follow-up appointment for infants diagnosed with moderate and severe BPD and for patients who are anticipated to be discharged with diuretics.

##### The NICU Team Is Aware of BPD and Labels ICD-10 Accordingly.

 Weekly communication of the pathway’s availability via the NICU medical director’s emails was the most effective method. The weekly emails identified infants reaching CGA of 36 weeks that should be screened for BPD based on their birth age (<32 wk) and their need for respiratory support. The communication directed the responsible providers to the guideline and later to the Care Signature Pathway (postimplementation) for proper evaluation and accurate coding of ICD-10 (P27.1).

##### Families Are Educated on BPD Management and Follow-up if Required.

 During the formal pediatric pulmonology consultation, the medical team engages with families, providing education about BPD. This includes detailed discussions on the necessity of outpatient follow-up and informing them about the heightened risk of hospital readmission within the first year after discharge. The initial visit is scheduled within 45 days of discharge, with subsequent appointments determined by patient characteristics and the pediatric pulmonologist’s decision.

#### Resources

##### Appointment Is Available within 45 Days of Discharge and Appointment Reminder.

 The pulmonary team scheduled a follow-up appointment before hospital discharge with the help of administrative staff. The pulmonary administration team actively reminded patients/guardians of their appointments, and a centralized call center delivered an SMS text a few days before the appointment. If a patient cancels their appointment, it will be rescheduled to the next available appointment, per the family’s convenience, while trying to keep it within 45 days to meet our goals.

### Measures and Analysis

The primary outcomes were the percentage of patients diagnosed with moderate or severe BPD with a pulmonary follow-up appointment scheduled within 45 days of the initial hospital discharge and all patients with moderate or severe BPD, regardless of appointment scheduling, who successfully attended these follow-up appointments. Each month, we reviewed the medical records of infants included in our discharged list, cross-referencing it with the weekly communication emails from the medical director to ensure that no infants with BPD were missed. Using the dataset of patients identified who might meet the criteria for BPD, we reviewed whether they had been evaluated for BPD at 36 CGA and if the diagnosis and severity documented in the medical record were consistent with the 2019 definition of BPD by Jensen et al.^5^ Statistical process control charts were used to track our progress. We considered special cause variation (SCV) to have occurred when there were at least eight consecutive points above or below the centerline. The charts were developed using Microsoft Excel QIMacros (KnowWare Int, Denver, Co.).

### Ethical Considerations

This project was designed to improve clinical practice and processes at our institute. Based on quality improvement criteria, it was exempt from institutional review board review.

## RESULTS

During the baseline period (January 2021–December 2021), we identified 85 patients with ICD-10 codes suggestive of respiratory disease. Overall, eight distinct ICD-10 codes were identified. Six patients had expired, and 10 were transferred to another healthcare facility. Of the remaining 69 patients, 41 met the 2019 criteria for BPD, 20 patients had the correct ICD-10 code [0.49; 95% confidence interval (CI), 0.33–0.64], and 27 of 41 met the criteria for moderate or severe BPD. The severity classification was not documented in the EMR for these patients (**Figure 1, Supplemental Digital Content 1**, which describes Baseline data period and the intervention period data from January 2021 to September 2023, http://links.lww.com/PQ9/A561).

During our intervention period (January 2022 and September 2023), we identified 141 patients; nine expired, and 20 were transferred to another healthcare facility. Between January 2022 and September 2022, 48 met the 2019 criteria for BPD, and 25 patients had the correct ICD-10 code (0.52; 95% CI, 0.38–0.67). The severity classification for these patients was not documented in the EMR. Twenty-five patients met the criteria for moderate or severe BPD (**Supplemental Digital Content 1,** http://links.lww.com/PQ9/A561). After implementing the BPD pathway (September 2022), 53 of 56 patients had the correct ICD-10 code (0.95; 95% CI, 0.89–0.99), and 52 had severity classifications documented in the EMR. Forty-four patients met the criteria for moderate or severe BPD (**Supplemental Digital Content 1,** http://links.lww.com/PQ9/A561).

**Fig. 5.**
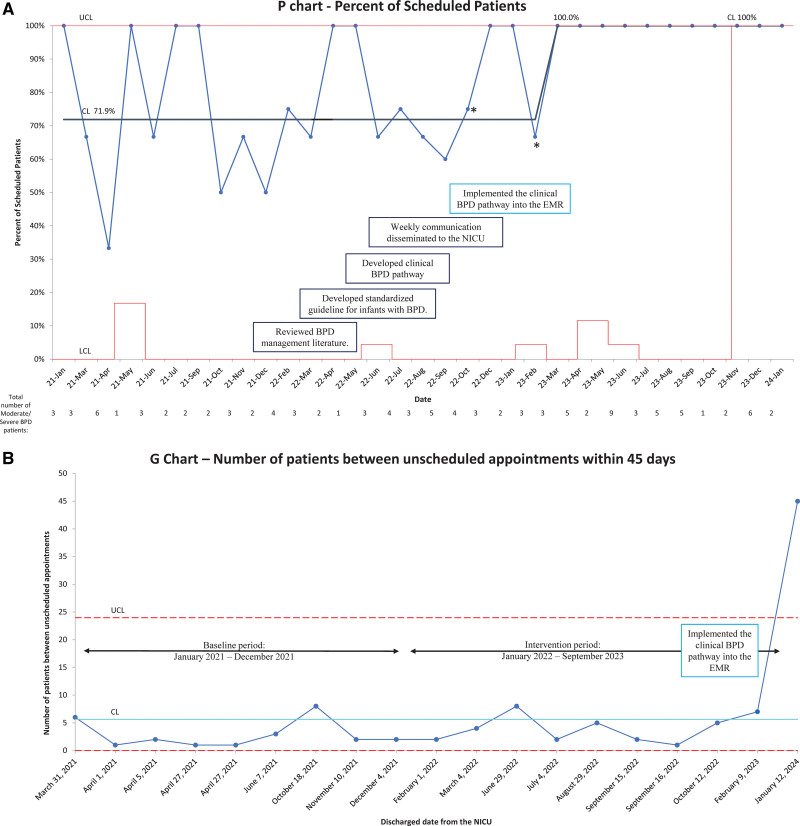
Statistical process control chart for appointment scheduling. A, Percent appointments scheduled within 45 days of discharge for moderate/severe BPD. Statistical process control chart, January 2021 to January 2024. *Two patients who did not receive a follow-up appointment as their ICD-10 code was mistakenly altered and the time the BPD pathway was revised. B, The number of scheduled patients between unscheduled patients or patient appointments more than 45 days after discharge. Statistical process G chart, January 2021 to January 2024.

The three patients with an incorrect ICD-10 code were assessed when they reached 36 weeks of age and were accurately diagnosed following the appropriate pathway. However, upon discharge, their ICD-10 codes were mistakenly altered. Consequently, due to the erroneous change in their ICD-10 codes, two patients (marked with * in Fig. 5A) did not receive a follow-up schedule within 45 days of discharge.

**Fig. 6.**
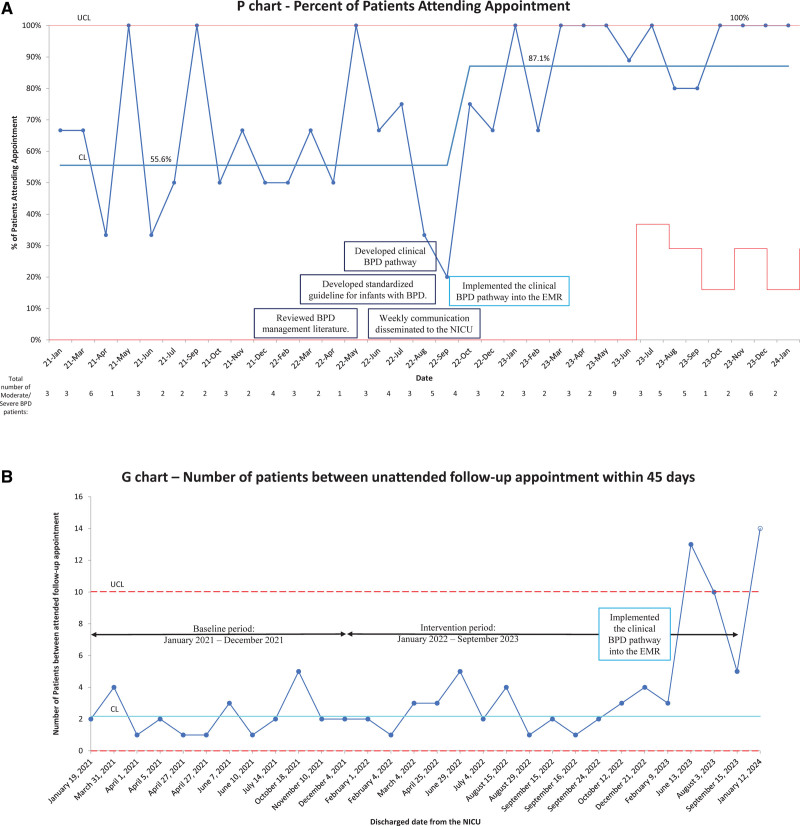
Statistical process control chart for appointment attendance. A, Percent attendance of scheduled appointments within 45 days for moderate/severe BPD. Statistical process control chart, January 2021 to January 2024. B, The number of scheduled patients attending clinic between those not attending a follow-up appointment from discharge date for moderate/severe BPD. Statistical process G chart, January 2021 to January 2024.

Figures 5A and 6A depict the proportion of infants with moderate and severe BPD who were scheduled for and completed a pulmonary follow-up appointment within 45 days of discharge each month, respectively. Figure 5B depicts the number of scheduled patients between unscheduled patients or those without a pulmonary follow-up appointment within 45 days of discharge. Figure 6B depicts the number of scheduled patients who attend a pulmonary follow-up appoint between those who did not.

During the baseline period, 71.9% were scheduled for follow-up 45 days after discharge. We achieved SCV in March 2023. After our interventions were completed, 100% were scheduled for follow-up 45 days after discharge (Fig. 5A).

During the baseline period, the overall attendance rate remained at 55.6%. With the implementation of our interventions, we achieved SCV in October of 2022, with an attendance rate of 87.1% for premature babies with moderate or severe BPD (Fig. 6A).

The BPD pathway was utilized 574 times from September 2022 to September 2023.

## DISCUSSION

Our study found significant variability in the diagnostic, therapeutic, and care practices for BPD among healthcare providers, even within the same healthcare facility, which can result in inconsistent management and follow-up for affected patients. Our baseline data revealed opportunities for improvement in current practices, including the absence of standardized assessment of premature infants with BPD, inconsistent engagement with pediatric pulmonologists, and the establishment of postdischarge follow-up meetings as routine components of care. By implementing six interventions directed at three key drivers, we increased our percentage of patients scheduled for follow-up appointments to 94.9% and the attendance rate to 87.1%. This improvement was observed following the introduction of our Care Signature BPD pathway.

Interventions centered on clinical pathway development were impactful in reaching our aim. The pathway successfully provided standardized knowledge, evidence-based best practices, and resources to NICU physicians and nurses in a user-friendly and timely format. After implementing the Care Signature BPD pathway, we observed SCV in the diagnosis, classification, follow-up, and attendance rate of patients with BPD.

All patients we identified using the methods described earlier and after implementing the BPD pathway met the 2019 criteria^5^ for BPD, and 95% were assigned the correct ICD-10 code. Most notably, 93% of the patients received appropriate severity classifications, indicating improved consistency in assessment.

During our monthly discharge list review, it appeared that the three patients assigned an incorrect ICD-10 code had undergone accurate assessment and diagnosis once they reached 36 weeks of age. These patients experienced an extended hospitalization period not associated with respiratory issues, and their ICD-10 code was mistakenly removed under the assumption that their BPD diagnosis had been resolved. These incidents prompted a revision of the BPD pathway. This change highlighted the importance of maintaining the BPD diagnosis and scheduling follow-up appointments with pediatric pulmonologists, even if the patient no longer required respiratory support or exhibited no respiratory problems. This revision led to the SCV, which we observed in the schedule rate in March 2023. Because that change in the pathway, every patient with a BPD diagnosis has been scheduled for a follow-up appointment with the pediatric pulmonology team.

Along with increasing our rates of scheduled and attended appointments, we demonstrated a decrease in variability, indicating a higher degree of consistency and predictability in our processes. The results of this study may suggest significant improvement in follow-up practices for infants with moderate or severe BPD after discharge from the NICU. The NICU team, consisting of physicians and nurses, was briefed on the control charts, recognizing and commending their dedicated efforts while illustrating the project’s progress. The pulmonology department was also presented with the control charts during the annual QI meeting. By utilizing Statistical process control charts to monitor the process, healthcare providers can ensure high-quality patient care and continuously improve their practices.

Implementing standardized protocols, guidelines, and pathways can enhance patient safety, reduce variability in care delivery, improve adherence to evidence-based practices, and ultimately result in better clinical outcomes for pediatric patients.^13–17^ As available treatments and therapies continue to improve, the population of surviving premature infants is only predicted to increase.^10^ With more consistent pulmonary follow-up, patients with moderate and severe BPD may have improved long-term pulmonary outcomes. We believe that the Care Signature BPD pathway, education, and improved communication were the primary drivers that improved care. However, further studies with larger sample sizes and longer follow-up periods are warranted to validate our findings and assess the long-term impact of the Care Signature BPD pathway on patient outcomes.

Our study had two notable limitations. First, our study was limited to a single center, which could impact the generalizability of our results to other healthcare settings with different patient populations or care practices. Further studies involving multiple centers and larger sample sizes are needed to validate the effectiveness of the Care Signature BPD pathway in different settings. Second, our study only assessed short-term outcomes, and the long-term impact on patient outcomes remains to be determined. Further research with longer follow-up periods is needed to assess the sustained effectiveness of the pathway in improving patient outcomes.

Although our study did not directly assess balancing measures, it is crucial to recognize their significance in understanding our findings comprehensively, which could serve as a limitation to our study. Factors such as user fatigue from EMR reminders, appointment availability in pulmonology providers’ schedules, and the consult burden on the pulmonology team should be considered essential considerations. Although not explicitly measured in our study, we suggest that future research explore these potential balancing measures for a more nuanced and thorough analysis of the implications of our interventions.

## CONCLUSIONS

Our study highlights the potential benefits of implementing a standardized pathway for diagnosing, classifying, and follow-up of patients with BPD. It provides valuable insights for improving the care of these vulnerable infants in our setting and beyond.

Additionally, our study revealed that timely scheduling of a pulmonary follow-up appointment before discharge for moderate and severe BPD patients increased the likelihood of patients receiving care in a pulmonary clinic. Further investigation is required to assess the efficacy of pulmonary follow-up and hospital readmission rates attributed to respiratory-related morbidity.

## Supplementary Material


