# Supplementary material for: Quality Improvement Initiative Enhances Outpatient Pediatric Pulmonology Follow-up for Premature Infants with Bronchopulmonary Dysplasia
Source: Pediatr Qual Saf. 2024 Jun 7;9(3):e736. doi: 10.1097/pq9.0000000000000736 (PMC11161285; doi:10.1097/pq9.0000000000000736)
Supplement: Supplementary file 1 [file pqs-9-e736-s001.pdf]

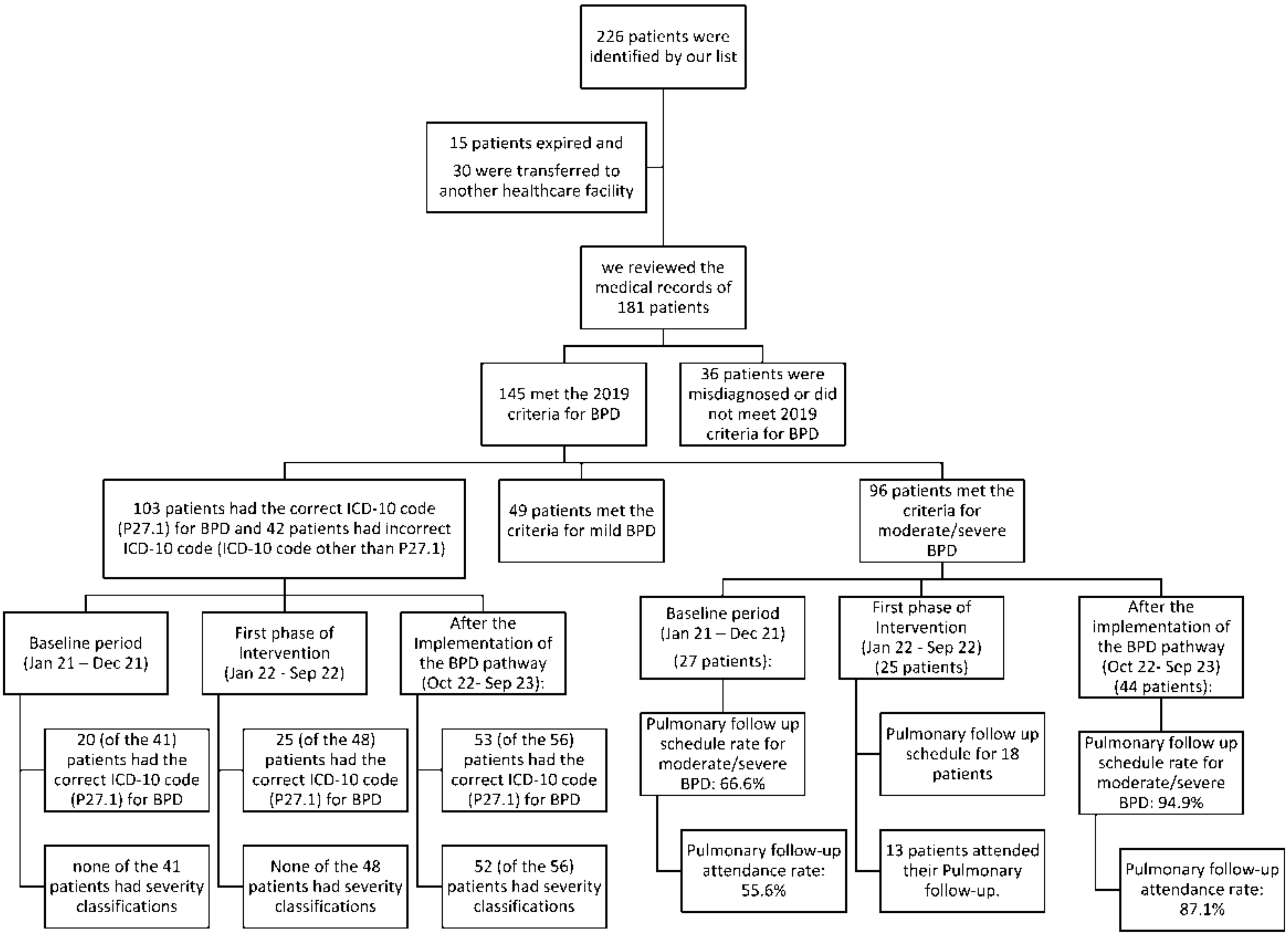

Supplemental Figure 1: Baseline data period and the interventions period data. January 2021 to September 2023.
